# Comparative Interpretation of CT and Standard Radiography of the Pleura

**DOI:** 10.5334/jbr-btr.1229

**Published:** 2016-11-19

**Authors:** Bart Ilsen, Frederik Vandenbroucke, Cathérine Beigelman-Aubry, Carola Brussaard, Johan de Mey

**Affiliations:** 1 UZ Brussel, BE

**Keywords:** Chest Radiography, CT, Pleura

## Abstract

Many diseases affect the pleural space in both adults and children, including common diseases such as pneumonia, cancer and heart failure. Pleural effusion is the most common manifestation of pleural disease, and it is often a secondary effect of another disease process.

Imaging plays a crucial role in the management of pleural disease. Chest radiography often remains the first examination in the assessment of these patients. Depending on the clinical context, the optimal imaging technique for further evaluation might be computed tomography (CT), ultrasound (US), or magnetic resonance (MR).

## Introduction

The pleura and pleural cavity are essential for efficient functioning of the lung, as the pericardium and pericardial cavity are for the heart. Pleural diseases represent a frequent problem in routine clinical practice, representing 25 percent of pulmonary unit consultations. The chest radiography remains the imaging modality of choice for the initial investigation of pleural disease. The use of US, CT, and MRI are therefore tailored to the patient and the clinical question.

In this article, we explore the pathologic manifestations of some conditions that primarily or secondarily affect the pleura by comparing the chest radiography and CT findings side by side. Nevertheless, this comparison between chest radiography and CT suffers the major drawbacks that views are mainly obtained in an upright position for the former and always in a recumbent position for the latter, which makes a strict comparison of pleural diseases difficult, as their aspects are frequently position dependent.

## Anatomy

The pleura is made of two serosal membranes, one covering the lung (the visceral pleura) and one covering the inner chest wall (the parietal pleura). Their surfaces glide over each other, facilitating proper lung movements during various phases of respiration. The transition between the parietal and visceral pleura is located at the pulmonary hilum. At this level, the reflection covers the hilum, except inferiorly, where the reflection extends down to the diaphragm and is called the triangular of inferior pulmonary ligament. They both consist of a single layer of flattened mesothelial cells that are subtended by layers of fibroelastic connective tissue. The connective tissue component of the visceral pleura is part of the peripheral interstitial fiber network and contains small vessels and lymphatic branches. The pleura receives its vascular supply from and is drained by the systemic circulation [1].

## Basic Imaging Principles

**Figure 1:**
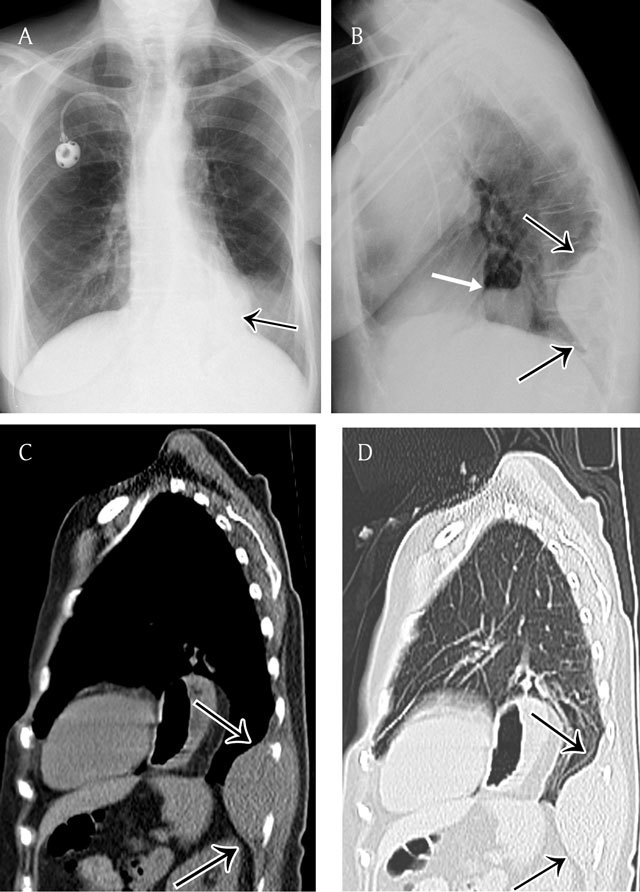
Extrapleural opacity. Hodgkin’s lymphoma of the rib cage – Frontal chest radiograph **(a)** shows a homogeneous retrocardiac opacity with sharp borders (black arrow). Lateral chest radiograph **(b)** shows a posterior opacity, presenting with obtuse angles (black arrows). Note the hiatel hernia (white arrow). A sagittal reformatted image in mediastinal **(c)** and lung window **(d)** shows the posterior mass and the rib destruction.

**Figure 2:**
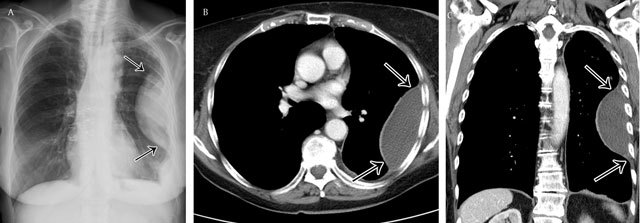
Pleural opacity. Encapsulated pleural effusion – Frontal chest radiograph **(a)** shows a lenticular opacity with smooth borders and obtuse angles (black arrows). Corresponding axial **(b)** and coronal **(c)** reformatted images demonstrate clearly the encapsulated pleural effusion.

**Figure 3:**
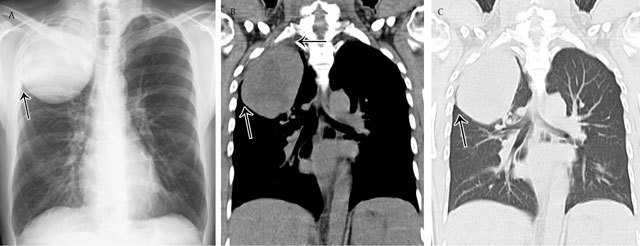
Parenchymal opacity. Adenocarcinoma of the right upper lobe – Frontal chest radiograph **(a)** shows a large mass in the right upper lobe presenting acute angles (black arrow) with the pleura and chest wall. Corresponding coronal reformatted CT image in mediastinal **(b)** and lung window **(c)**.

A peripheral opacity can be located in three different locations related to the pleura: (a) the extrapleural, (b) the pleural, and (c) the subpleural areas of the lung. An extrapleural opacity (Figure 1) originates from the chest wall, and when it does not invade into the pleura and lung, it presents with obtuse angles and a sharp medial margin related to the pleura. A pleural-based opacity (Figure 2) has margins that are partially or completely well circumscribed, indicating contiguity with the pleural surface and usually also presenting with obtuse pleural angles. A subpleural opacity (Figure 3) is located in the parenchyma and usually has irregular margins and acute pleural angles.

The standard chest radiograph may not permit accurate localization of focal lesions to the pleural space. General features of pleural tumors that have been described include a peripheral location abutting the chest wall, a sharp margin with the contiguous lung, and a tapering of obtuse angles with the rib cage of mediastinum.

## Pleural Effusion

Pleural effusion is the accumulation of fluid in the pleural space that results when forces that control the inflow and outflow of the space are disrupted [2]. According to their composition, most pleural effusions can be classified into two categories: transudate and exudate. Pleural transudate is a clear fluid with a protein content of less than 3 g/dl. Pleural exsudate is a more opaque fluid with a protein content of more than 3 g/dl. In general, transudates reflect a systemic perturbation (and therefore are commonly bilateral), whereas exudates usually signify an underlying local (pleuro-pulmonary) disease. The more common causes of transudative effusion are congestive heart failure and hypoalbuminemic states (e.g., cirrhosis), while those of exudative effusions are malignancy, infections (e.g., pneumonia), and inflammatory diseases. Other sorts of pleural effusions include hemothorax, chylothorax, pancreatic, bilious, and cerebrospinal fluid pleural effusions. The appearance of an effusion depends on the patient’s position and mobility of effusion (free or constrained to variable extent) at time of acquisition.

### Distribution of Pleural Effusion in the Erect Patient

**Figure 4:**
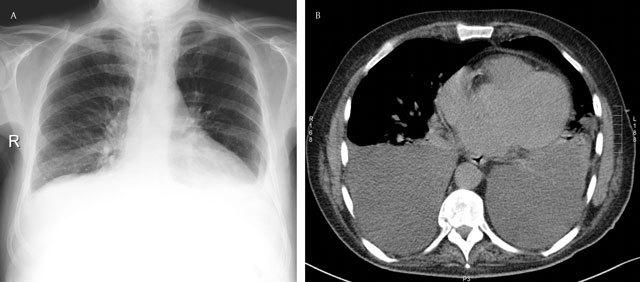
Subpulmonary effusion – Frontal chest radiograph **(a)** shows an elevation of the left and right hemidiaphragm (“pseudo-diaphragm). There is an obliteration of the intrapulmonary blood vessels. Note the blunting of both costophrenic sulcus. The gastric air bubble is not seen. Corresponding axial CT image **(b)**.

The distribution of free pleural fluid depends on the patient’s position, as it moves in dependent position due to gravity. In the upright position, the initial site of fluid accumulation is between the base of the lung and the diaphragm, namely, the subpulmonic region (Figure 4). This represents a diagnostic challenge on posteroanterior and lateral chest radiograph as the upper edge of the fluid mimics the contour of the diaphragm and results only in a pattern of slight hemidiaphragm elevation (“pseudo-diaphragm”)[4]. As the amount of fluid increases, there may be flattening and some inversion of the diaphragm without significant blunting of the lateral costophrenic angle.

Because the posterior costophrenic sulcus is the deeper part of the pleural cavity, relatively large amounts of pleural fluid may accumulate without being apparent on the upright posteroanterior view. The accumulation of 200 mL or more of pleural fluid usually leads to blunting of the lateral costophrenic sulcus, although sometimes up to 500 mL or even more may be present without any blunting [4]. As it better demonstrates the posterior costophrenic sulcus, the lateral view is more sensitive for detection of small pleural effusion than the frontal view.

When the amount of pleural fluid increases, the typical concave, upward-sloping contour of free fluid on erect frontal and lateral radiographs is known as the meniscus appearance. Fluid collects at the base of the pleural space due to gravity. The combination of positive hydrostatic intrapleural pressure at the lung base on the one hand and the elastic recoil of the lung on the other hand acts to force some fluid to rise against gravity and surround the lower part of the lung. As the X-ray beam must penetrate a greater depth of fluid at the periphery of the thorax, the upper margin at periphery appears higher [2,3,4]. Large pleural effusions obscure the contour of the heart and eventually displace the mediastinum contralaterally.

### Distribution of Pleural Effusion in the Supine Patient

**Figure 5:**
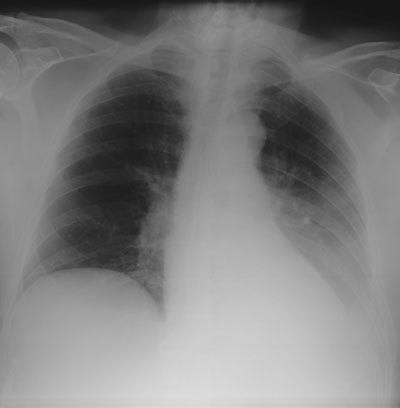
Subpulmonary effusion – Frontal chest radiograph shows a more lateral position of the right “pseudo-diaphragm”, being situated near the junction of the middle and lateral thirds. A correlation with CT is in this case impossible.

In the supine patient, free pleural fluid layers posteriorly and produces a hazy increase in opacity without obscuration of the bronchovascular markings, which may be difficult to detect, particularly when bilateral [5]. This homogeneous opacity may occupy only the lower part of the pleural cavity, making the lower half of the hemithorax more opaque than the upper (Figure 5). Blunting of the lateral costophrenic sulcus in the supine position occurs when the amount of fluid is sufficient to fill the posterior hemithorax up to the level of the sulcus [6]. Other signs include obscuration of the hemidiaphragm, apparent elevation of the hemidiaphragm, decreased visibility of the lower love vessels below the level of the apparent dome of the diaphragm, increased opacity of the spleen in left-sided effusion, and thickening of the minor fissure [6,7].

Free pleural fluid can also cap the apex of the lung on supine radiographs, known as the apical cap sign. Due to the small volume and capacity of the apex, this is considered to be an early sign, as fluid extends tangentially to the X-ray beam to a greater degree between the lung and chest wall at the apex than at the base [2].

### Atypical Distribution and Loculation of Pleural Fluid

**Figure 6:**
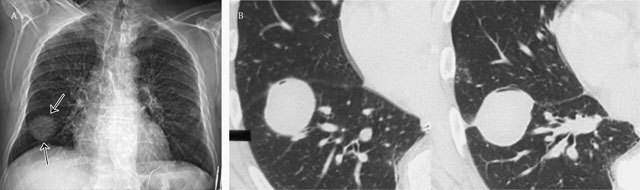
Vanishing tumor or pseudotumor – Frontal chest radiograph **(a)** shows a mass at the right lung base presenting smooth borders (black arrows). Corresponding axial CT image (2 consecutive images) in lung window **(b)** clearly demonstrates the encapsulated fluid in the major fissure simulating a tumor.

**Figure 7:**
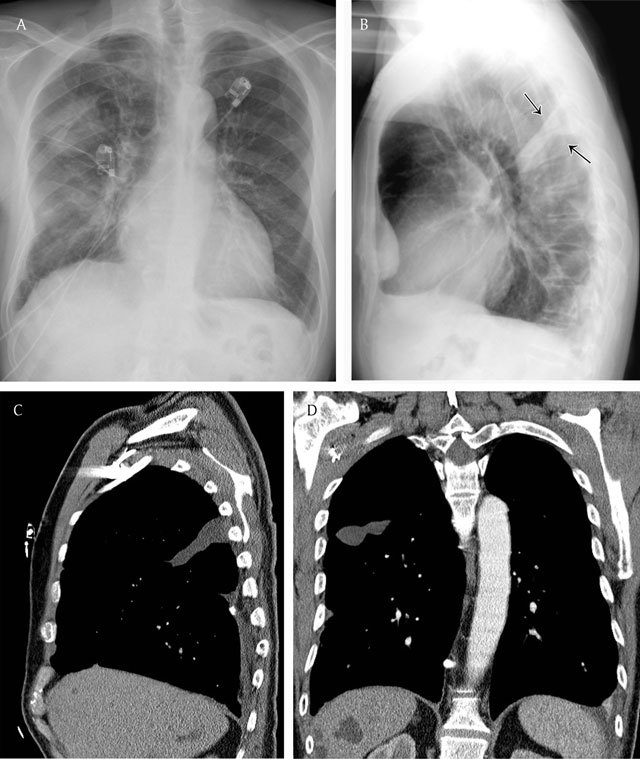
Lenticular configuration of loculated fluid in the fissure – Frontal chest radiograph **(a)** shows a hazy increase in opacity, not sharply defined in the upper part of the right hemithorax. Lateral chest radiograph **(b)** shows the loculated fluid in the major fissure (black arrows), confirmed on sagittal **(c)** and coronal **(d)** reformatted images on CT.

Loculation of pleural effusion can occur when adhesions between contiguous surfaces of the pleura develop, often in the case of pyothorax or hemothorax. When it occurs between two lobes, it can be misdiagnosed as a pulmonary neoplasm on chest radiographs. However, fluid accumulations between two lobes tend to absorb spontaneously and therefore have been called “vanishing tumor” or pseudotumor (Figure 6). Fluid loculated in a fissure has a distinctive lenticular configuration on the lateral view, allowing differentiation from condensation or atelectasis (Figure 7). When the elastic recoil of the lung is restricted, the retractility of the lung is modified, and the pleural fluid is attracted towards this area [8]. Therefore, it must be kept in mind that any atypical distribution of pleural fluid may be a sign of both parenchymal and pleural disease.

## Empyema

Thoracic empyema, or pyothorax, is defined as pus in the pleural cavity. The majority of empyemas follow acute bacterial pneumonias or lung abscesses. Other causes include thoracic surgery, trauma, mediastinitis, and spread from extrapulmonary sites, such as osteomyelitis of the spine and cervical or subphrenic abscess. Empyemas are associated with an inflammatory pleural reaction, and when the infection is inadequately treated, it may lead to a pleural fibrosis.

**Figure 8:**
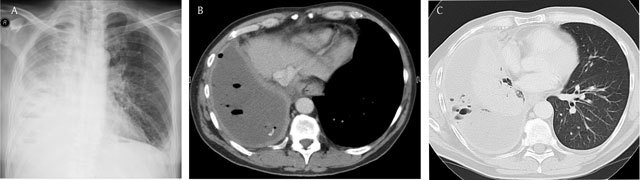
Empyema in a patient with cough and fever since 1 week – Chest radiograph **(a)** demonstrates a hazy increased opacity with multiple air bubbles overlying the lower lung. An axial contrast-enhanced CT view in mediastinal **(b)** and lung window **(c)** at the level of the lung base shows thickening and an enhancement of the parietal pleura and the visceral pleura (split pleura sign). Note the multiple air bubbles in the empyema.

**Figure 9:**
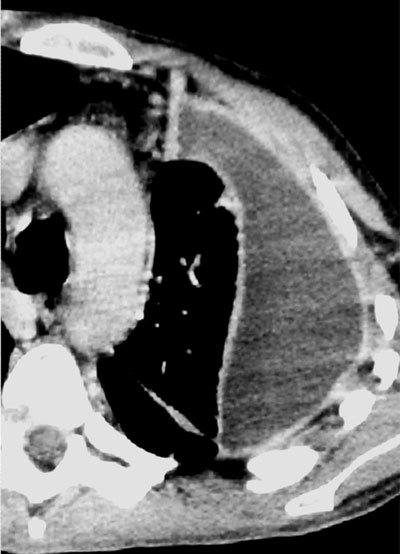
Split pleura sign – An axial contrast-enhanced CT view in mediastinal window at the level of the lung basis shows a pleural effusion with an enhancement and thickening of the visceral and parietal pleura.

Pleural fluid is often present and usually unilateral, but when bilateral, it is substantially greater in volume on the infected side [9]. Similar to sterile effusions, non-loculated empyemas are homogeneous in opacity, change with patient position, and have a meniscus sign. When loculated, empyemas should be differentiated from lung abscess, which may be difficult but has important therapeutic consequences. Differentiation is more easily achieved using CT than chest radiograph. The presence of enhancement of the parietal and visceral pleura, thickening of the extrapleural subcostal tissues, and increased attenuation of the extrapleural fat should suggest an empyema (Figure 8). The combination of fluid between the pleural space and the thickening of the visceral and parietal pleura is referred to as the split pleura sign (Figure 9)[10].

## Asbestos-related Pleural Disease: Pleural Plaques

The pleura is thought to be more sensitive to asbestos than the lung parenchyma. Pleural disease can occur as pleural effusion, pleural plaques, or diffuse pleural thickening. Pleural plaques are the most common manifestation of asbestos fibers inhalation and are well-known as an indicator of a previous exposure. The prevalence of pleural plaques correlates with the intensity of asbestos exposure and the time interval from its initial exposure. The latency period between exposure to asbestos and development of pleural plaques is approximately 15 years; for radiologically visible calcified pleural plaques, the latency period is at least 20–30 years [11,12].

Pleural plaques are discrete circumscribed areas of hyaline fibrosis of the parietal pleura and rarely the visceral pleura. They consist of mature collagen fibers arranged in an open basket-weave pattern and are covered by flattened or cuboidal mesothelial cells. The plaques mainly involve the posterior and anterolateral aspects of the pleura, following the contours of the posterolateral 7th to 10th intercostal spaces. They spare the lung apices and costophrenic angles and rarely extend vertically for more than four interspaces. They almost always involve only the parietal pleura (and are therefore asymptomatic) but have rarely been described in the interlobar fissures.

**Figure 10:**
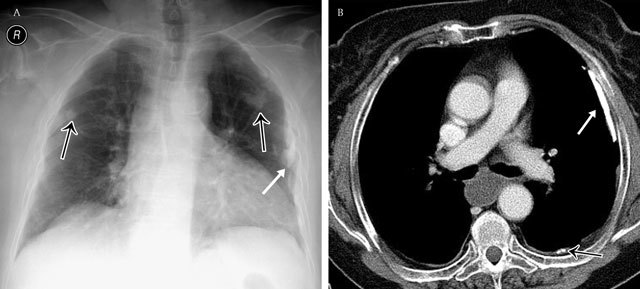
Asbest-related pleural plaques in a 59 year-old builder – Frontal chest radiograph **(a)** shows leaf-like or “geographic” opacities when the pleural plaques are viewed en face (black arrows). Tangentially viewed, pleural plaques are seen as focal areas of pleural thickening (white arrows). Corresponding axial CT image **(b)**.

Chest radiography has been the primary radiographic method for the detection of asbestos-related pleural disease for a long time. When viewed en face, pleural plaques may be difficult to recognize unless they are large or calcified and are recognized as multiple and bilateral nodular, stippled, irregular, leaflike, or “geographic” opacities (Figure 10). Plaques within fissures can mimic solitary pulmonary nodules. When viewed tangentially, pleural plaques are seen on chest radiographs as focal areas of pleural thickening. Over the diaphragm, they produce either curvilinear calcifications or scalloping.

Chest radiography however has a relatively low sensitivity for the detection of asbestos-related pleural diseases, and new techniques such as dual-energy digital subtraction chest radiography may improve detection [13]. Another potential source of difficulty when reading conventional radiographs is the misinterpretation of extrapleural fat as pleural thickening. Subcostal fat may mimic pleural thickening in obese individuals. Typically, it appears as a symmetrical, smooth, and sometimes undulating soft-tissue density. It typically extends from the 4th to the 8th ribs.

CT has a greater sensitivity and specificity for identifying asbestos-related pleural diseases than conventional radiography [14,15]. Pleural plaques appear as well-circumscribed areas of pleural thickening separated from the underlying rib and extrapleural fat by a thin layer of fat. These plaques can look like “table mountains” or mesas. They can have a nodular configuration and can impinge slightly on the adjacent lung parenchyma. This results in a focal hypoventilation, which may lead to the formation of a pulmonary subpleural curvilinear line adjacent to the plaque.

## Diffuse Pleural Thickening

Diffuse pleural thickening is defined as an extensive scarring of the pleura which results into thickening and fibrosis of the visceral pleura, with fusion to the parietal pleura. The response of the mesothelial cell to injury and the ability of it and the basement membrane to maintain their integrity is pivotal as to whether or not fibrosis occurs, and cytokines, growth factors and reactive oxygen species (ROS) are likely to play a crucial role [17].

Because of the fibrosis and fusion of visceral and parietal pleura, diffuse pleural thickening might have in contrast to pleural plaques, a clinical impact. They can cause thoracic pain and may be associated with a "constrictive" deficit in pulmonary function.

Diffuse pleural thickening may be caused by empyema, hemothorax, connective tissue disorder, or asbestos exposure [18].

Radiographically, it is considered to be present when a smooth, uninterrupted pleural opacity is seen extending over at least a fourth of the chest wall with or without obliteration of the costophrenic sulci [19] (Figure 14a).

**Figure 11:**
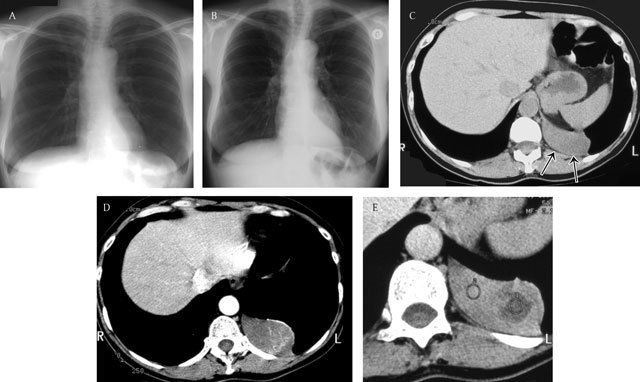
Localised fibrous tumor of the pleura – Two consecutive chest radiographs **(a, b)** with an interval of 3 years show a sharply delineated slow growing retrocardiac mass. Unenhanced axial CT image **(c)** shows a well-defined homogeneous soft-tissue-density mass. Note the intact subpleural fat (black arrows). Enhanced CT images **(d, e)** show heterogeneous enhancement corresponding to areas of necrosis. (Courtesy of G. Ferreti, Grenoble, France).

**Figure 12:**
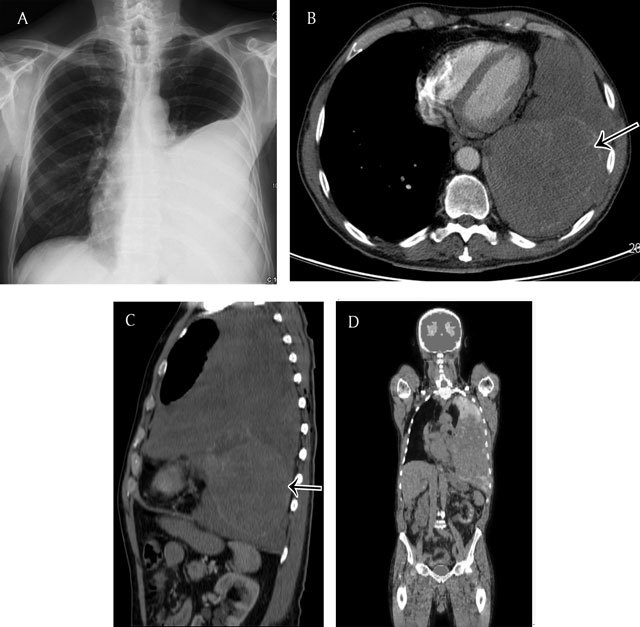
71-year old man with dyspnea and dry cough since 3–4 months. Chest radiograph **(a)** shows a large left pleural effusion. Axial **(b)** and sagittal **(c)** CT image of the chest demonstrates a soft tissue mass occupying the left pleural space. Note the enhancing component at the lower part of the mass (black arrows). Body PET-CT (fluoro-deoxyglucose) **(d)** shows a hot spot located at the upper part of the pleural tumor. Pathology revealed a giant solitary fibrous tumor (partially hypermetabolic on PET-CT) of the pleura with sarcomatous transformation (enhancing component).

**Figure 13:**
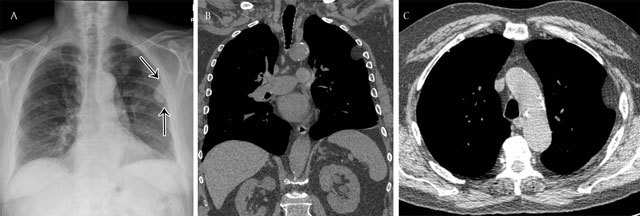
Pleural lipoma in a 79-year old male – Frontal chest radiograph **(a)** shows a homogeneous opacity with smooth margins, a sharply defined medial edge, and an ill-defined lateral border (black arrows). A coronal reformat CT view in mediastinal window **(b)** clearly shows the pleural based lesion **(b)**. The axial CT view **(c)** shows the smooth marginated lesion forming obtuse angles with the chest wall. Note the fat attenuation of the pleural-based lesion.

**Figure 14:**
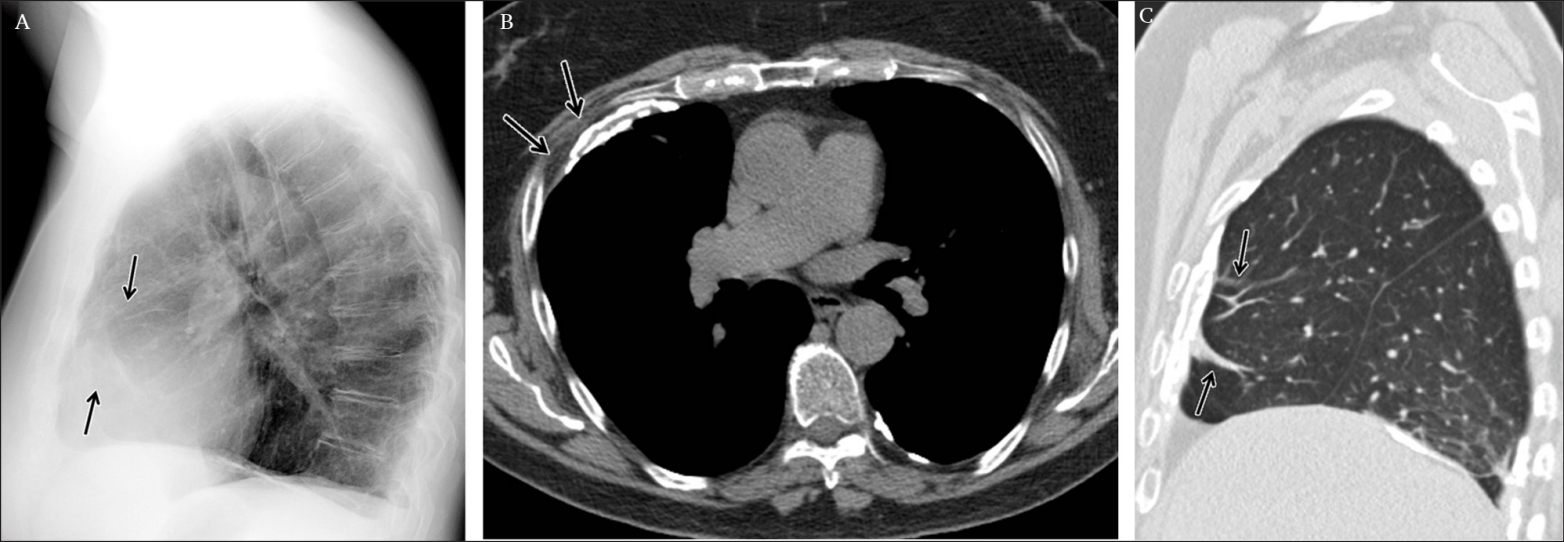
Diffuse Pleural Thickening in a 63-year old man with a history of asbestos exposure – Lateral chest radiography **(a)** shows a smooth uninterrupted pleural opacity extending over at least a fourth of the anterior chest wall. Note some small linear opacities in the adjacent lung parenchym(arrows). Axial CT image in soft tissue window **(b)** demonstrates a partially calcified pleural thickening associated with a hypertrophy of the subpleural fat (arrows). Sagittal CT image in lung window **(c)** shows parenchymal bands adjacent to the pleural thickening as a result from the fusion of the two pleural layers.

As with discrete pleural plaques, CT is more sensitive and specific for the detection of diffuse pleural thickening than chest radiography. On CT they can be defined as a contiguous sheet of pleural thickening more than 5 cm wide on transverse CT images, more than 8 cm in extent in craniocaudal images and more than 3 mm thick [19]. They might be calcified and are often associated with a hypertrophy of the subpleural fat (Figure 14b). The adjacent lung parenchym is affected and this is manifected by parenchymal bands and rounded atelectasis. Parenchymal bands are linear opacities 2–5cm in length, sometimes shorter, extending from the pleural thickening through the lung. When they radiate from the surface of the pleural thickening they can give the appearance of “crow’ feet” (Figure 14c)

## Localised Fibrous Tumor of the Pleura

A localized fibrous tumor of the pleura is a slow-growing primary pleural neoplasm unrelated to asbestos exposure. It is a relatively uncommon neoplasm with benign features [16] that accounts for less than 5 percent of all pleural tumors. Approximately 80 percent arise from the visceral pleura, and the majority presents as pedunculated mass. Due to great diversity of histologic findings, it is known by a variety of terms, including benign localized mesothelioma, benign pleural fibroma, fibrosing mesothelioma, and pleural fibromyxoma. It has been described in all age groups but has a peak incidence in persons older than 50 years of age.

The radiographic appearance depends on the size of the tumor. A small to medium-sized tumor usually appears as a solitary, homogeneous, sharply delineated, often lobulated nodule or mass arising from the visceral pleura that forms obtuse angles with the chest wall (Figure 11). Large tumors can appear as an opacity occupying a considerable portion of one hemithorax and can lose their obtuse angle with the chest wall. In the case of a pedunculated tumor, the determination of an extrapulmonary origin may not be possible on the chest radiograph. The stalk can be as long as 9 cm, allowing the lesion to move according to the patient’s position.

CT imaging shows a well-delineated and often lobulated mass [16]. On unenhanced CT scans, they appear with soft-tissue attenuation. Calcification can be present in large tumors and is related to areas of necrosis. Contrast enhancement can be homogeneous and intense as a result of the rich vascularization of the tumor. However, heterogeneous enhancement can be seen due to necrosis and the myxoid degeneration of hemorrhage within the tumor [16,17]. Tumors larger than 10 cm are subject to malignant transformation (Figure 12) and usually demonstrate central necrosis. Nevertheless, there are no definite radiologic features to differentiate benign tumors from malignant fibrous tumors; therefore, resection is advised in all patients.

## Pleural Lipoma and Liposarcoma

Pleural lipomas are rare tumors, usually asymptomatic, and found incidentally on the chest radiograph. Lipomas may be intrathoracic or transmural, with an intrathoracic and extrathoracic component. They can be accurately diagnosed at CT, as they appear as a well-defined mass of homogeneous fat attenuation presenting with obtuse angles related to the chest wall and displacing the adjacent pulmonary parenchyma (Figure 13). Liposarcoma is an even more uncommon tumor that usually has a heterogeneous mixture of fat and soft-tissue attenuation.
